# Impact of mental health and addiction NIMHANS ECHO on primary care physicians: study from a rural state of India

**DOI:** 10.1192/bjo.2021.438

**Published:** 2021-06-18

**Authors:** Shabinabegam A M Sheth, Bhavya Bairy, Aurobind Ganesh, Sumi Jain, Prabhat Chand, Pratima Murthy, Sanjeev Arora

**Affiliations:** 1 Central and North West London NHS Foundation Trust, Dept of Psychiatry, National Institute of Mental Health and Neurosciences; 2 Dept of Psychiatry, National Institute of Mental Health and Neurosciences; 3 State Health Society; 4 Project ECHO, University of New Mexico Health Science Centre

## Abstract

**Aims:**

As per National Mental Health Survey-2015-16, 83 out of 100 people having mental health problems do not have access to care in India. Further, primary health care providers (PCPs) have not been adequately trained in the screening, diagnosis, and initial management of common mental health conditions. There is thus a need to train health care providers at the State level to incorporate mental health into primary health care. In this paper, we report the findings of a collaborative project between the National Institute of Mental Health and Neuro Sciences (NIMHANS) Bangalore India, and the state of Chhattisgarh incorporating mental health into primary care and addressing urban-rural disparities through tele-mentoring.

**Method:**

We assessed the impact of the NIMHANS Extended Community Health Care Outcome (ECHO), an online, blended training program on participants' knowledge and competence (primary outcome) and commitment, satisfaction, and performance (Secondary outcomes) using Moore's evaluation framework. Primary and secondary outcomes were determined through a pre-post evaluation, assessment of trainee participation in the quarterly tele ECHO clinic as well as periodic assignments, respectively.

**Result:**

Over ten months of the NIMHANS ECHO program, there was a significant improvement in the participants' knowledge post-ECHO (p < 0.05, t = −3.52). Self-efficacy in diagnosis and management of mental health problems approached significance; p < 0.001. Increased engagement in tele-ECHO sessions was associated with better performance for declarative and procedural knowledge. The attrition rate was low (5 out of 30 dropped out), and satisfaction ratings of the course were high across all fields. The participants reported a 10- fold increase in the number of patients with mental health problems they had seen, following the training. A statistically significant increase in the number of psychotropic drugs prescribed post ECHO with t = −3.295, p = 0.01.

**Conclusion:**

The outcomes indicate that the NIMHANS ECHO with high participant commitment is a model with capacity building potential in mental health and addiction for remote and rural areas by leveraging technology. This model has the potential to be expanded to other states in the country in providing mental health care to persons in need of care.

